# Widespread Recombination Suppression Facilitates Plant Sex Chromosome Evolution

**DOI:** 10.1093/molbev/msaa271

**Published:** 2020-10-23

**Authors:** Joanna L Rifkin, Felix E G Beaudry, Zoë Humphries, Baharul I Choudhury, Spencer C H Barrett, Stephen I Wright

**Affiliations:** 1 Department of Ecology and Evolutionary Biology, University of Toronto, Toronto, ON, Canada; 2 Centre for Analysis of Genome Evolution and Function, University of Toronto, Toronto, ON, Canada

**Keywords:** sex chromosome, plants, recombination, *Rumex*, gene density

## Abstract

Classical models suggest that recombination rates on sex chromosomes evolve in a stepwise manner to localize sexually antagonistic variants in the sex in which they are beneficial, thereby lowering rates of recombination between X and Y chromosomes. However, it is also possible that sex chromosome formation occurs in regions with preexisting recombination suppression. To evaluate these possibilities, we constructed linkage maps and a chromosome-scale genome assembly for the dioecious plant *Rumex hastatulus*. This species has a polymorphic karyotype with a young neo-sex chromosome, resulting from a Robertsonian fusion between the X chromosome and an autosome, in part of its geographic range. We identified the shared and neo-sex chromosomes using comparative genetic maps of the two cytotypes. We found that sex-linked regions of both the ancestral and the neo-sex chromosomes are embedded in large regions of low recombination. Furthermore, our comparison of the recombination landscape of the neo-sex chromosome to its autosomal homolog indicates that low recombination rates mainly preceded sex linkage. These patterns are not unique to the sex chromosomes; all chromosomes were characterized by massive regions of suppressed recombination spanning most of each chromosome. This represents an extreme case of the periphery-biased recombination seen in other systems with large chromosomes. Across all chromosomes, gene and repetitive sequence density correlated with recombination rate, with patterns of variation differing by repetitive element type. Our findings suggest that ancestrally low rates of recombination may facilitate the formation and subsequent evolution of heteromorphic sex chromosomes.

## Introduction

Plant and animal genomes vary widely in recombination rate, both between and along chromosomes (Gaut et al. 2007), but how this variation contributes to genome evolution remains unclear. Natural selection can favor the invasion of variants that change the rate of recombination when those variants reduce recombination between coadapted alleles (Charlesworth B and Charlesworth D 1973). However, selection is not the only factor that affects the evolution of recombination rates. Chromosomal position (Haenel et al. 2018), chromatin structure (Ohta et al. 1994), and gene and transposable element density (Tian et al. 2009; Kent et al. 2017) all influence rates of recombination, and evolutionary changes in these properties may indirectly drive recombination rate evolution. Nonetheless, compelling evidence supporting a role for natural selection in the evolution of recombination rates is growing (Rieseberg et al. 1995; Noor et al. 2001; Ortiz-Barrientos et al. 2016; Samuk et al. 2020). Disentangling the relative contributions of selection and other factors in shaping the rate of recombination is essential for detailed understanding of the forces shaping genome evolution.

Sex chromosomes are particularly valuable for the study of recombination rate evolution because they represent an example of convergent recombination suppression, and evolutionary theory predicts an important role for natural selection in this process. Classical models of sex chromosome evolution predict that sex chromosomes evolve from autosomes to alleviate the cost of sexually antagonistic alleles in the sex to which those alleles are deleterious (Charlesworth D and Charlesworth B 1980; Rice 1984). Because of differences between the sexes in their optimal reproductive strategies (Trivers 1972), some alleles beneficial in one sex can be detrimental in the other sex, creating a selective load in the population (Lande 1980; Rice 1984). The cost of this genetic load can be resolved by the evolution of sex-specific gene expression (Mank 2009), or by the invasion of recombination modifiers that link the sexually antagonistic variant with the genomic region responsible for determining the sex in which that variant is beneficial (Rice 1987). Depending on dominance and the strength of selection (Kidwell et al. 1977; Lenormand 2003; Otto 2019), sexually antagonistic selection can promote the spread of structural rearrangements, including inversions and autosome–sex chromosome fusions (Charlesworth D and Charlesworth B 1980; Rieseberg 2001), and cause recombination loss to spread further along the X and Y (Bull 1985). Over time, according to these models, recombination suppression spreads in a stepwise fashion along an incipient sex chromosome, leaving a pattern of “strata” with distinct levels of divergence between the X and Y. Evidence for evolutionary strata on sex chromosomes has been found in diverse organisms, including humans (Lahn and Page 1999), chickens (Handley et al. 2004), and the plant *Silene latifolia* (Bergero et al. 2007). Because plant sex chromosomes generally have recent origins and are more likely to be in earlier stages of divergence than vertebrate sex chromosomes, they can be particularly useful for understanding the initial conditions driving sex-chromosome formation and the associated evolution of recombination rates.

*Rumex hastatulus* (section Americanae: Navajas-Pérez et al. 2005) is one of a small number of dioecious (i.e., with separate male and female individuals) plants with heteromorphic sex chromosomes (Charlesworth 2013). This species offers a powerful model for the study of sex chromosome evolution because of its polymorphic sex chromosome karyotype (Smith 1964). Males to the west of the Mississippi River have four autosomes and an XY pair (the “XX/XY cytotype”), whereas males to the east of the Mississippi River have three autosomes, a single larger X chromosome, and two Y chromosomes (the “XX/XY_1_Y_2_ cytotype”). Historical and contemporary cytological studies (Smith 1964, 1969; Grabowska-Joachimiak et al. 2015; Kasjaniuk et al. 2019) strongly suggest that this sex chromosome polymorphism arose from a reciprocal translocation and the loss of heterochromatic segments through dysploid reduction (hereafter simplified as “fusion”; see Schubert and Lysak 2011) between the ancestral X chromosome and an autosome. The current polymorphic karyotype of the species includes both the shared ancestral and the derived state of this chromosome. *Rumex hastatulus*, therefore, provides an unusual opportunity to investigate recombination rates in homologous chromosomes before and after linkage to the sex-determining region. The principal goal of our study was to understand the changes in recombination rate associated with sex chromosome turnover in *R*. *hastatulus* and to relate our results to the classic model of sex chromosome evolution.

Here, we present a chromosome-scale genome assembly of *R. hastatulus* and describe the patterns of recombination and genome content on the sex chromosomes and autosomes. With these data, we investigate: 1) whether the pattern of recombination rate heterogeneity and polymorphism is consistent with the classic stepwise model of sex chromosome evolution, 2) how recombination rates evolve following the formation of a neo-sex chromosome, and 3) how recombination rate correlates with genome content on sex chromosomes and autosomes.

## Results

### The Ancestral Sex-Linked Region Exhibits Low Sex-Averaged Recombination Rates

#### Genome Assembly and Linkage Map

For the genome assembly, we sequenced one male individual from the XX/XY cytotype using PACBio (see Materials and Methods). Following de novo assembly of PACBio SMRTcell reads, contigs were assembled into longer scaffolds using in vitro reconstituted chromatin Chicago libraries (Putnam et al. 2016), followed by Hi-C chromosome conformation capture libraries (Lieberman-Aiden et al. 2009; Belton et al. 2012). This primary assembly (“primary assembly”; archived on Dryad https://doi.org/10.5061/dryad.s7h44j14h) comprised 1.647 Gb of the *R. hastatulus* genome, with half of the genome assembled into 25 scaffolds larger than 11.886 Mb (N50). Our assembly size is consistent with C-value estimates for *R. hastatulus* based on flow cytometry from both previous work (Kasjaniuk et al. 2019) and the present study (supplementary table S1, Supplementary Material online). Based on these values, the primary assembly represents approximately 92% of the estimated total genome size.

**Fig. 1.**
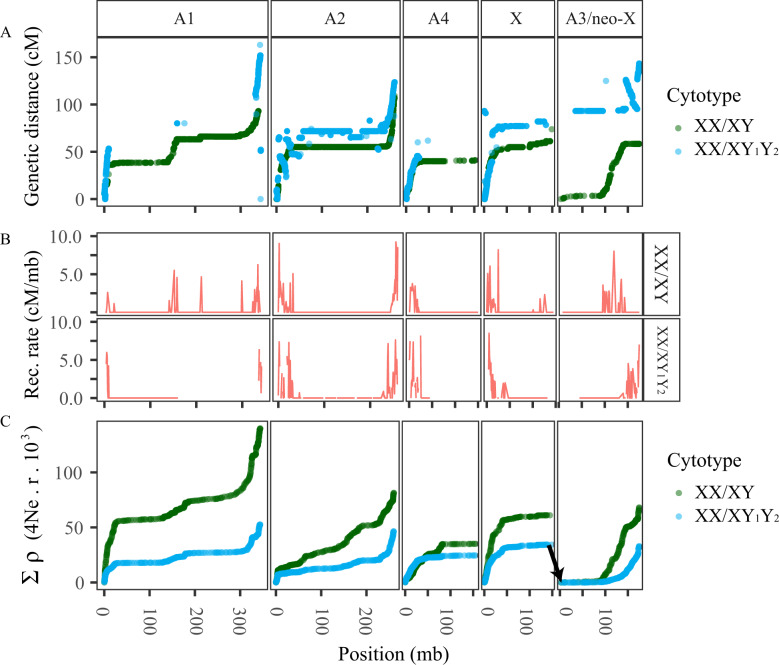
The recombination landscape across the chromosomes of *Rumex hastatulus*. (*A*) Marey map relating linkage map sex-averaged recombination position (cM) to physical genetic position in the XX/XY-cytotype genome assembly (Mb; panels are XX/XY-cytotype chromosomes) for the XX/XY cytotype (green) and XX/XY_1_Y_2_ cytotype (blue). (*B*) Sex-averaged recombination rate (cM/bp), estimated as coefficients of linear models for 1Mb windows. (*C*) Cumulative sex-averaged recombination rate (ρ) estimated from population-level genetic sequencing for the XX/XY cytotype (green) and XX/XY_1_Y_2_ cytotype (blue). Cumulative recombination estimates have been reset to start at zero at the border of the XX/XY_1_Y_2_-cytotype neo-sex chromosome to facilitate direct comparison with the XX/XY-cytotype A3. Black arrow shows linkage between X and A3 (the XX/XY_1_Y_2_-cytotype neo-sex chromosome) in the XX/XY_1_Y_2_-cytotype, which has been repositioned on A3 for more direct comparison between the cytotypes.

Linkage mapping allowed for further scaffolding and correction of misjoins to assemble the genome into the expected five major scaffolds, representing the five chromosome pairs of the XX/XY cytotype of *R. hastatulus* (Smith 1964). Using RNAseq from 96 F_2_ individuals of both sexes (192 total; see supplementary table S2, Supplementary Material online, for sex information and methods for linkage mapping information), we constructed the final genetic map from 988 independent markers on five linkage groups (supplementary table S3, Supplementary Material online). The five chromosomal scaffolds (“secondary assembly,” not including unincorporated scaffolds, archived on Dryad: https://doi.org/10.5061/dryad.s7h44j14h and COGE: https://genomevolution.org/coge/GenomeInfo.pl?gid=58357, last accessed November 3, 2020) comprised a total of 1.08 Gb, or 65% of the primary assembly. The karyotype of the XX/XY-cytotype *R. hastatulus* includes two large metacentric chromosomes and three smaller chromosomes variously described as metacentric, submetacentric, and heterobrachial (Smith 1964; Bartkowiak 1971; Grabowska-Joachimiak et al. 2015). Patterns of recombination along the chromosomes are consistent with these centromere locations: Three chromosomes (the X, A3, and A4) have a single region of high recombination at one end and are presumably submetacentric or acrocentric, whereas two (A1 and A2) have high-recombination regions at both ends and an area of suppressed recombination in the center, and thus are likely metacentric (fig. 1*A* and supplementary fig. S1, Supplementary Material online).

#### Identification of the Ancestral Sex Chromosome

**Fig. 2.**
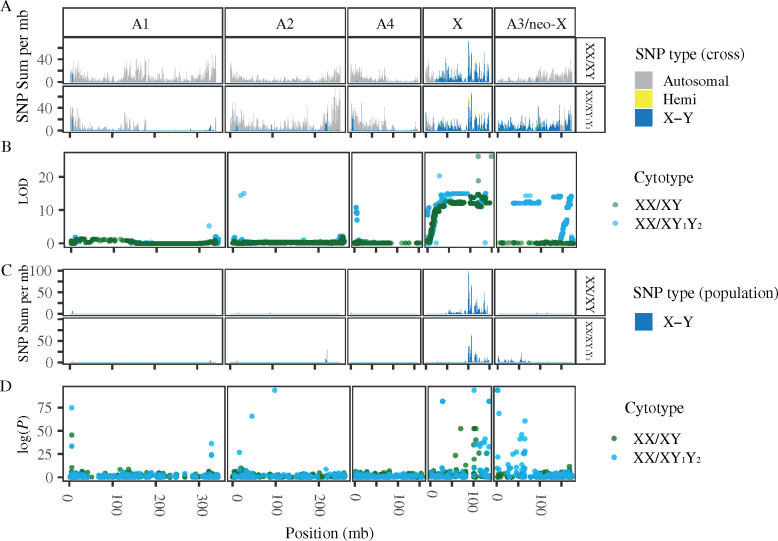
Association and linkage to sex across the genome of *Rumex hastatulus.* (*A*) Sum of SNPs identified in sliding 500-kb windows as autosomal (gray), hemizygous (yellow), and X- or Y-linked (dark blue) for the XX/XY cytotype (top panel) and XX/XY_1_Y_2_ cytotype (bottom panel) in cross data from (Hough et al. 2014). (*B*) QTL analysis of sex as a binary trait, for the XX/XY cytotype (green) and XX/XY_1_Y_2_ cytotype (blue). (*C*) Sum of SNPs identified in sliding 500-kb windows as hemizygous (yellow), and X-Y linked (dark blue) for the XX/XY cytotype (top panel) and XX/XY_1_Y_2_ cytotype (bottom panel) in population-wide data from (Hough et al. 2014; Beaudry et al. 2020). (*D*) GWAS for association with sex in population GBS data from (Beaudry et al. 2020) for the XX/XY cytotype (green) and XX/XY_1_Y_2_ cytotype (blue).

Using our chromosome-level assembly, we first used existing RNAseq data from an XX/XY-cytotype within-population cross (Hough et al. 2014) to identify single nucleotide polymorphisms (SNPs) showing X-linked, Y-linked, X-hemizygous, and autosomal segregation patterns. Interestingly, only 52% of SNPs whose inheritance patterns suggest sex linkage were mapped to any of the major chromosomal scaffolds, indicating that a significant proportion of the sex chromosome sequence could not be positioned in our linkage map, but was present in our assembly as smaller scaffolds. In contrast, 84% of SNPs with autosomal inheritance patterns were mapped to the major scaffolds; this suggests that the X chromosome assembly is less complete, likely due to a combination of a high frequency of heterozygous sites in the mosaic haploid assembly of X- and Y-linked regions of the sex chromosome pair, and to high repeat and/or heterochromatin density on the Y (Grabowska-Joachimiak et al. 2015). Of the SNPs located on the five major chromosomal scaffolds, one linkage group contained 97% of SNPs classified as showing Y-linked segregation patterns and 96% of SNPs classified as showing X-linked segregation patterns (supplementary table S4, Supplementary Material online; fig. 2*A*). Based on this, we identified that linkage group as the shared, and therefore ancestral, sex chromosome and hereafter refer to it as the X (or XY pair). With a quantitative trait locus (QTL) mapping approach, all markers with locations past 9.78 Mb on the X chromosome were significantly associated with sex phenotype (*P *<* *0.01, fig. 2*B*). Thus, overall, we have identified a very large (>140 Mb) sex-linked region comprising the vast majority of the X chromosome. The SNP segregation patterns and QTL analyses also suggested the presence of a pseudoautosomal region where recombination occurs at one end of the chromosome (figs. 1*A* and 2*B*).

As cross-based analysis may not capture rare recombination events between the X and Y chromosomes, we also used population-level sequence data (Hough et al. 2014; Beaudry et al. 2020) to assess the boundaries of the sex-linked region. Using RNA-sequencing data from population samples in a previous study (Hough et al. 2014), we identified sites as likely to be fully sex-linked when all males were heterozygous and all females were homozygous. The vast majority of such sites were located on the chromosome previously identified as the XY pair (supplementary table S5, Supplementary Material online). The region of X–Y fixed differences from the population-based data was slightly narrower than from the crossing data, suggesting that this approach did indeed capture more recombination events (fig. 2*C*). We also conducted a genome-wide association study (GWAS) for sex phenotype using population-level genotyping-by-sequencing (GBS) data (Beaudry et al. 2020). We found that the vast majority of SNPs with significant association with sex, after Bonferroni correction (*P *<* *4.42* *×* *10^−5^), were located on the X (fig. 2*D*). As these multiple, largely independent approaches identified the identical region of the same chromosome as showing a significant association with sex, we conclude that we have effectively identified the X chromosome.

#### Suppressed Recombination on the Shared Sex Chromosome

Sex-averaged recombination rate estimates for the sex-linked region were very low across a large region of the chromosome (<0.01* *cM/Mb; fig 1*B* and supplementary table S2, Supplementary Material online). To quantify this, we conducted windowed analysis of recombination rates (fig. 2*B*) and fit linear models to the relation between recombination and base pair distance using 1-Mb windows. Negative estimates were converted to 0* *cM/Mb, as they are reflective of low resolution due to very low recombination, low marker density, and/or finite numbers of F_2_ mapping individuals. Consistent with the presence of a large region of very low recombination, 75% of the windows along this chromosome showed recombination rate estimates <0.01* *cM/Mb. This is particularly prominent in the sex-linked region. After the first 20 Mb of the chromosome, containing the pseudoautosomal region, 93% of windows in the sex-linked region had very low recombination rate estimates.

Although our crossing design restricts us to a sex-averaged recombination rate estimate, the low rate we find on the X cannot simply reflect suppressed recombination between the X and the Y in males. Even if male recombination in the sex-linked region is 0, we still infer that 93% of genomic windows in the sex-linked region have female recombination rates <0.02 cM/Mb. In contrast, 75% of windows in the first 20 Mb had rates >0.5 cM/Mb, implying a major suppression of recombination in the sex-linked region on the X.

To determine whether this pattern of low recombination was detectable at the population level, we estimated ρ, the sex-averaged effective recombination rate (4 *N*_e_*r*), by applying LDhat (Stumpf and McVean 2003; Auton and McVean 2007) to our population-level RNAseq polymorphism data (Hough et al. 2014; Beaudry et al. 2020). It is important to note that population-based estimates of the population recombination parameter can be affected by both recombination rate and differences in effective population size (Kuhner et al. 2000). These can be influenced by the strength of linked selection, which in turn is affected by recombination rates and the density of functional sites (Charlesworth 1998; Auton and McVean 2007; Beissinger et al. 2016; Haenel et al. 2018). Nevertheless, the large-scale patterns we detected are consistent with the linkage map data and suggest very low effective rates of recombination in the sex-linked region (fig. 1*C*). Because the low rate should reflect both X–X and X–Y recombination, these results further suggest that recombination is generally low in this genomic region. Low rates of recombination are common in regions surrounding the centromeres of many organisms (Mahtani and Willard 1998), and our findings are therefore consistent with the possibility that the sex-linked region in *R. hastatulus* is within a very large pericentromeric region.

We observed considerable heterogeneity along the X chromosome in the density of sex-linked SNPs, a proxy for X–Y differentiation (fig. 2*A* and *C*). Although this is consistent with previous gene-level results in *R. hastatulus* (Hough et al. 2014), which also identified varying levels of divergence across transcripts possibly consistent with strata, a clear stepwise pattern is not apparent from the chromosome scale. To further assess the possibility of strata, we used BLAST to align the transcripts from Hough et al. (2014) to this assembly and examined divergence along the chromosome. The X–Y *K*_S_ values, ordered along the X chromosome, showed no definitive stepwise pattern (supplementary fig. S2, Supplementary Material online). Given the difficulty with fully ordering and assembling this region of low recombination and the fact that a significant fraction of the X scaffolds remain unplaced, our results should be considered inconclusive as to the likelihood of strata on the X.

### The Neo-Sex-Linked Region Also Has Low Recombination Rates

To determine whether low rates of recombination are a widespread feature of sex chromosomes in *R. hastatulus*, we also investigated the recombination rate on the neo*-*X of the XX/XY_1_Y_2_ cytotype. We constructed a linkage map using 877 independent markers for the XX/XY_1_Y_2_ cytotype, complementary to the XX/XY-cytotype map (fig. 1*A* and supplementary fig. S1, Supplementary Material online). Recombination patterns along our genetic map indicate that the linkage groups represent three metacentric chromosomes and one submetacentric chromosome. This is consistent with the reduction in chromosome count expected following the X–autosome fusion in this cytotype (Smith 1964). A single large metacentric linkage group in the XX/XY_1_Y_2_-cytotype linkage map joined the XX/XY-cytotype X chromosome with the XX/XY-cytotype chromosome A3, suggesting that A3 in the XX/XY cytotype is the autosomal homolog of the neo-X chromosome. Although the smallest autosome, A4, was originally believed to be the source of the fused chromosome, more recent hybridization-based approaches instead identify A3 (Kasjaniuk et al. 2019), which is consistent with our findings. From previous work, the position of a ribosomal DNA repeat cluster suggests that in the translocation event leading to the fusion, the longer arm of A3 was translocated to the sex chromosome and the shorter arm was lost (Kasjaniuk et al. 2019). Our linkage map also identified a large inversion on the recombining end of the neo-X, as well as a linked pair of inversions on one arm of A2 (fig. 1*A*). The inversion on the neo-X appears to have extended the region of low recombination in this karyotype (supplementary fig. S1, Supplementary Material online). However, given that this inversion lies in the pseudoautosomal region (see below) it may not be linked to sex, and it is not possible to conclude whether this inversion affects recombination between the neo-X and neo-Y without a sex-specific linkage map.

Using cross data from the XX/XY_1_Y_2_ cytotype from Hough et al. (2014), we identified X- and Y-linked SNPs on both the ancestral (panel X) and neo-X (panel A3) sections of the large fused X chromosome (fig. 2*A* andsupplementary table S4, Supplementary Material online). At the population level, we observed fixed X–Y differences on both the ancestral and neo-X, but the region of fixed X–Y differences on the ancestral X was less extensive than in the XX/XY cytotype (fig. 2*C*). QTL analysis (fig. 2*B*, P < 0.01) and GWAS (fig. 2*D*, *P *<* *5.19* *×* *10^−05^) for sex association in the XX/XY_1_Y_2_ cytotype identified large regions on both the X and A3 chromosomes, overlapping the regions showing low rates of recombination.

In common with the X chromosome of the XX/XY cytotype, the fused chromosome exhibited a very large nonrecombining region derived from both the shared X chromosome and the neo-X. In particular, 93% of 1-Mb windows corresponding to the sex-linked region had recombination estimates <0.01* *cM/Mb. In contrast, only 32% of the first 20 Mb and 48% of the last 30 Mb of the chromosome, corresponding to the two pseudoautosomal regions, showed similarly low recombination rates. As with the shared sex chromosome, suppressed male recombination alone cannot account for this degree of difference in recombination rate between the sex-linked and pseudoautosomal regions.

Recombination rates on the neo-X were similar to that of the ancestral autosome in the XX/XY cytotype, including a 92.16-Mb region of 5.682* *cM (averaging 0.074* *cM/Mb) on A3 in the XX/XY cytotype (the homolog of the neo-X), where most 1-Mb windows showed very low rates of recombination (fig. 1*B*). This pattern accords with our population-level estimates of recombination rate (fig. 1*C*). Although A3 segregates independently from the sex chromosomes and shows no signal of sex linkage in the XX/XY cytotype (fig. 2), it has a low recombination rate comparable to the recombination rate of its fused homolog. However, the extent of recombination suppression appears to have increased and shifted following the fusion, likely due to both the chromosomal inversion and the loss of one of the chromosome arms (fig. 2*A* and *B*). Taken together, this evidence implies that strong recombination suppression in the majority of the genomic region that formed the neo-sex chromosome of the XX/XY_1_Y_2_-cytotype preceded its status as a sex chromosome, with an additional inversion potentially further extending the region of low recombination.

### Low Recombination Is a Genome-Wide Phenomenon

The sex chromosomes of *R. hastatulus* are not unusual in exhibiting low chromosome-wide recombination. Our analyses also revealed that all autosomes had massive (>100* *Mb) regions of minimal recombination in both cytotypes, with evidence for recombination restricted primarily to the tips of the chromosomes (fig. 1*A* and *B*). This finding from *R. hastatulus* is consistent with patterns observed from comparative data across plants and animals, which suggest that species with large chromosomes often have highly peripheral recombination (Haenel et al. 2018; Otto and Payseur 2019). *Rumex hastatulus* appears to represent an extreme case, with all chromosomes exhibiting over 100* *Mb with recombination rates near zero. Notably, the degree of recombination suppression on A3 in the XX/XY cytotype is not unusual compared with the other autosomes; using 1-Mb windows, 73% of this chromosome had windows with very low recombination rate estimates (<0.01* *cM/Mb), compared with 84.6% (A1), 84.3% (A2), and 74.5% (A4). These values were also comparable to the X chromosome (75%). Remarkably, overall approximately 81% of the genome exhibits very low recombination and, given that our linkage map contains only 65% of the complete genome and low-recombination sequence is more difficult to position and assemble, this value is likely an underestimate.

### Recombination Rate and Genome Content

**Fig. 3.**
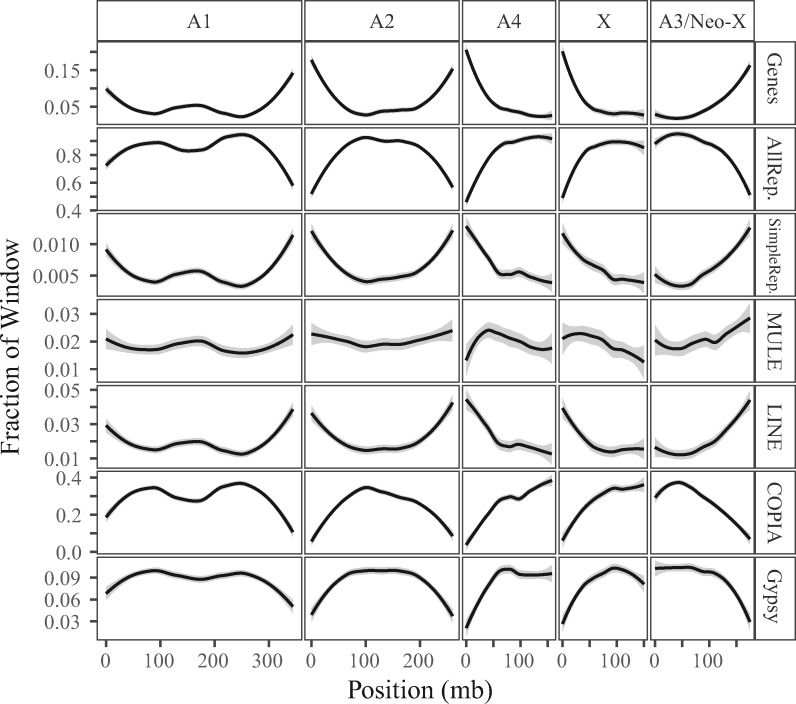
Genome content across the chromosomes of *Rumex hastatulus*, measured as proportion of 500-kb windows. Panels show repeat-filtered gene density, density of all repetitive elements (“AllRep.”), simple repeat density (“simpRep”), mutator-like element (MULE) density, LINE (long interspersed nuclear element) density, copia element density (COPIA), and *Gypsy* element density.

Recombination rate is known to correlate with both gene density and with the density of repetitive sequence (Tian et al. 2009; Kent et al. 2017). As previously observed in other plants, including *Arabidopsis*, rice, maize, and wheat (Wright et al. 2003; Dvorak et al. 2004; Anderson et al. 2005; Matsumoto et al. 2005), we found higher gene density in high-recombination regions (fig. 3 and supplementary fig. S3, Supplementary Material online). The nonrecombining region of the sex chromosome is gene-poor but similar to other low-recombination regions across the genome. The pattern for repetitive element densities varied by element class. Long terminal repeat retrotransposons were concentrated in low-recombination areas, whereas long interspersed nuclear elements and simple repeats were more common in high-recombination areas. Both protein-coding genes and repeats vary in density with the recombination landscape.

## Discussion

The major finding of this study is that the sex-linked regions of both cytotypes of *R. hastatulus* are embedded in vast regions of very low recombination which cannot be explained simply by a lack of recombination between the X and the Y chromosome. Rather, multiple lines of evidence suggest that recombination rates are low both before and after linkage with sex and that this pattern of low recombination is genome-wide.

### Implications for Predicting the Evolution of Sex Chromosomes

The classic model of sex chromosome evolution assumes that the invasion of recombination modifiers is subsequent to the appearance and maintenance of a sex-determining region and sexually antagonistic variants (Charlesworth D and Charlesworth B 1980; Rice 1987). However, the accumulation of recombination modifiers may not be necessary if the region already has low rates of recombination (Charlesworth 2018, 2019). Rather, in regions with low rates of recombination, the cost of the invasion and subsequent maintenance of sexually antagonistic variation on sex-linked regions is reduced. Indeed, low rates of recombination are predicted to increase the likelihood of the maintenance of sexually antagonistic variation on sex chromosomes that still recombine and, under certain conditions, on autosomes (Rice 1987; Fry 2010; Charlesworth 2019; Otto 2019). Thus, regions of the genome with low rates of recombination may be generally predisposed to evolve sex-linked regions (Charlesworth 2019). A role for ancestrally low rates of recombination in sex-chromosome evolution is consistent with evidence from other plant species, such as papaya, *Carica papaya* (Iovene et al. 2015), and kiwifruit, *Actinidia chinensis* (Pilkington et al. 2019). Analogously, self-incompatibility alleles, which also benefit from recombination suppression (Kamau and Charlesworth 2005), have been identified in a region of low recombination close to a centromere in *Petunia* (ten Hoopen et al. 1998). By comparing a neo-sex chromosome with its ancestral autosome, we have provided key evidence suggesting that suppressed recombination was the ancestral state prior to the evolution of sex linkage.

The vast regions of low recombination on the sex chromosomes of *R. hastatulus* are part of a genome-wide pattern of recombination-suppressed chromosomes and may predispose taxa with such genome organization to evolve heteromorphic sex chromosomes. Simulations suggest that high heterogeneity in recombination rate can maintain more genetic diversity at loci under opposing selection than homogeneous recombination rates (Berner and Roesti 2017). Although recombination rates have not yet been quantified in other *Rumex* species, it is noteworthy that sex chromosomes have arisen only in sections of the genus with reduced chromosome numbers (Navajas-Pérez et al. 2005), consistent with repeated chromosomal fusions. Widespread recombination suppression across the genomes of *Rumex* species may have contributed to the independent evolution of heteromorphic sex chromosomes in another clade of *Rumex*, especially given that the sex chromosomes of species in *Rumex* section Acetosa (such as *R. rothschildianus* and *R. acetosa*) are unlikely to be homologous to either of the *R. hastatulus* sex chromosomes (Navajas-Pérez et al. 2005; Quesada del Bosque et al. 2011; Crowson et al. 2017). Although *Rumex* may represent an extreme case, large chromosomes with extensive regions of suppressed recombination and asymmetry in chromosome arm lengths are common in flowering plants, and the conditions necessary for this kind of sex chromosome fusion may occur in other species with sex chromosomes (Stebbins 1971; Haenel et al. 2018). Across many systems, recombination appears to be more strongly concentrated near telomeres in males than in females, which may further facilitate the evolution of sex chromosomes from pericentric sex-determining regions (Sardell and Kirkpatrick 2020). Overall, our results provide further support for the proposal that sex chromosome evolution may be strongly influenced by genome organization.

### Synthesis with Previous Studies in *Rumex*

Our finding that in *R. hastatulus* low recombination is likely ancestral to the evolution of sex and neo-sex chromosomes may seem at odds with two earlier findings in this species: 1) Previous estimates of neutral genetic diversity indicated lower diversity on the Y but not on the X chromosome, which was consistent with much stronger effects of linked selection on the Y than on the X (Hough et al. 2017), yet here we suggest that recombination rates are likely low on both X and Y in this region; 2) given that ancestrally low recombination can remove the requirement for the invasion of recombination modifiers to relegate sexually antagonistic variation to the sex chromosomes, how can we also account for results demonstrating that genes with higher expression in the male haploid (pollen), which are strong candidates for sexual antagonism in plants, are disproportionately recruited to the ancestral Y chromosome (Sandler et al. 2018)?

There are two possibilities for the pattern of low neutral genetic diversity on the Y chromosome. A selective sweep may be responsible, or the low diversity may result from the nonlinear dynamics predicted in the transition from low rates of recombination to an absence of recombination. Selective sweeps can cause dramatically reduced neutral genetic variation in genomic regions under selection (Nielsen 2005), and are reported to be important in explaining low diversity on the Y chromosomes of fruit fly, *Drosophila melanogaster*, and papaya, *C. papaya* (Larracuente and Clark 2013; VanBuren et al. 2015). If a strongly favored Y haplotype swept through *R. hastatulus* populations, a pattern of reduced diversity would result and could also contribute to the low estimate of population-wide recombination rate (ρ = 4 *N*_e_*r*). Previous work estimating the strength of selection on the *R. hastatulus* Y chromosome found that while purifying selection was sufficient to explain the levels of genetic diversity, positive selection could play a role if few sites were under selection (Hough et al. 2017). We found low gene density in the sex-linked region of the Y (fig. 3*A*) and thus it is possible that fewer sites are under selection than were reported in previous estimates. With fewer sites under selection, selective sweeps on the Y could be important drivers of reduced diversity.

Additionally, even small differences in rates of recombination can have important effects on neutral diversity (Hartfield et al. 2010). For example, simulations suggest that a very low rate of recombination in the European common frog, *Rana temporaria*, can account for the maintenance of high genetic diversity on the Y (Rodrigues et al. 2018) and, similarly, of high genetic diversity across the genome of the largely asexual apomictic buttercup, *Ranunculus auricomus* (Hojsgaard and Hörandl 2015). In *R. hastatulus*, a low rate of recombination between X chromosomes in females may not bear the same consequences as a possible complete absence of recombination between X and Y, and the low rates of recombination between X chromosomes may be sufficient to maintain genetic diversity in this region. This would imply that the Y and possibly the neo-Y chromosomes have experienced additional recombination suppression. Further work estimating sex-specific recombination rates, and explicit model-fitting of these rates to patterns of neutral diversity, will help untangle the interplay of recombination, selection and diversity on both the X and Y chromosomes.

Initially low but nonzero rates of recombination are in fact likely to be the optimal parameter space for the invasion of recombination modifiers. Tight linkage facilitates the maintenance of sexually antagonistic polymorphism and, subsequently, the invasion of recombination modifiers on the sex chromosomes (Charlesworth D and Charlesworth B 1980). The very low, but nonzero, rates of recombination on the sex chromosomes demonstrated in our study are thus suitable for the maintenance of polymorphism along the proto-sex chromosomes. Low rates of recombination may allow for the invasion of recombination modifiers completely linking haploid-expressed and male-specific (pollen) genes to the Y, a process known as “pollenization” (Scott and Otto 2017; Sandler et al. 2018). Although the work of Sandler et al. (2018) suggests that ancestrally low rates of recombination along the proto-sex chromosome may have contributed to the subsequent pollenization of the Y, future tests for a preexisting enrichment of pollen-biased genes on Autosome 3 (neo-X) would provide further evidence for a role for haploid selection in the spread of recombination suppression.

Consistent with this idea, our results also suggest that additional recombination suppression has occurred on the neo-sex chromosome since the fusion. In particular, recombination rate suppression extends further, possibly due in part to the presence of a chromosomal inversion in the XX/XY_1_Y_2_-cytotype pseudoautosomal region (fig. 1). Inversions have been shown to be important in the evolution of sex chromosomes, as well as in ecological differentiation and reproductive isolation (Rieseberg et al. 1995; Noor et al. 2001; Huang and Rieseberg 2020; Todesco et al. 2020). In future work, we plan to identify the inversion’s breakpoints and explore the possibility of a role for selection and for inversions in the spread of recombination suppression along the sex chromosomes in *R. hastatulus*. Phased assemblies of the X and Y chromosomes will also help enable further assessment of the presence of evolutionary strata to investigate the possibility of a history of additional recombination suppression in these preexisting low-recombination regions.

Suppressed recombination may also have contributed to the likelihood of the chromosome fusion by allowing repetitive sequence to accumulate. Chromosome “fusions” result from reciprocal translocations initiated by ectopic recombination or double-strand break repair with ectopic homologous templates followed by loss of chromosome fragments (Schubert and Lysak 2011). The smaller chromosomes (A3 and A4) in *R. hastatulus* appear to have two uneven arms (Smith 1964; Bartkowiak 1971; Grabowska-Joachimiak et al. 2015), and we found that one arm of each was recombination suppressed, depleted of genes, and enriched in repetitive sequence (fig. 3). This repetitive sequence on A3 likely provided templates for the ectopic recombination leading to the “fusion,” and the loss of these segments may have been less costly because of their low gene content.

## Conclusions

Preexisting low-recombination may play a crucial role in sex-chromosome formation in plants (Charlesworth 2019). In both kiwifruit and papaya, sex-determining regions appear to have originated in centromeric regions that were likely ancestrally recombination-suppressed (Iovene et al. 2015; Pilkington et al. 2019). Although the sex chromosomes of papaya and kiwifruit are small and homomorphic or weakly heteromorphic, in *R. hastatulus* the extensive genome-wide scale of recombination suppression may have contributed to the ongoing formation of large, heteromorphic sex chromosomes. The ancestral recombination landscape may thus be a major determinant of the genomic structure of sex chromosomes.

## Materials and Methods

### Primary Genome Assembly

We sent 3 g of leaf tissue from an *R. hastatulus* male F_1_ from two parents from Wesley Chapel, TX (TX-WES in Pickup and Barrett [2013]) to Dovetail Genomics LLC, Santa Cruz, CA. At Dovetail, high molecular weight DNA was extracted and sequenced on 15 PacBio SMRTcells (Single Molecule, Real-Time; Eid et al. 2009). The sample was sequenced to 35× coverage for a total of 6.7M reads. After error correction, 5.7M reads were retained (24× coverage) with an N50 of 9.5 kb. The error-corrected reads were assembled by Dovetail Genomics into a primary assembly using Falcon (Chin et al. 2013, 2016), and the assembly was polished with Arrow from the PacBio GenomicConsensus toolkit (https://github.com/PacificBiosciences/GenomicConsensus, last accessed November 3, 2020). This assembly yielded 43,461 contigs with an N50 of 74.7 kb.

We sent an additional 3 g of leaf tissue from a full-sib male for two orders of further scaffolding by Dovetail Genomics to improve the primary PacBio-Falcon assembly. The assembly was first scaffolded using the Chicago technique (Putnam et al. 2016), which uses in vitro reconstituted chromatin for positioning. Two Chicago libraries were sequenced from ∼500 ng of high molecular weight gDNA reconstituted into chromatin in vitro and fixed with formaldehyde. Fixed chromatin was digested with DpnII, 5′ overhangs were filled in with biotinylated nucleotides, and free blunt ends were ligated. After ligation, crosslinks were reversed and the DNA purified from protein. Purified DNA was treated to remove biotin that was not internal to ligated fragments. The DNA was sheared to approximately 350 bp mean fragment size, and sequencing libraries were generated using NEBNext Ultra enzymes and Illumina-compatible adapters. Dovetail isolated biotin-containing fragments using streptavidin beads before polymerase chain reaction (PCR) enrichment of each library. Libraries were sequenced on an Illumina HiSeq X. The number and length of read pairs produced were: 189 million, 2 × 150 bp for library 1; 170 million, 2 × 150 bp for library 2. Together, these Chicago library reads provided 33.42× physical coverage of the genome (1–100 kb). These reads were used to scaffold the PacBio-Falcon assembly using the HiRise pipeline (Putnam et al. 2016) which is designed specifically for using proximity ligation data to scaffold genome assemblies. Dovetail conducted an iterative analysis. They aligned shotgun and Chicago library sequences to the draft input assembly using a modified SNAP read mapper (http://snap.cs.berkeley.edu, last accessed November 3, 2020). The separations of Chicago read pairs mapped within draft scaffolds were then analyzed by HiRise to produce a likelihood model for genomic distance between read pairs, and the model was then used to identify and break putative misjoins, to score prospective joins, and to make joins above a threshold. The longest scaffold increased from 600 to 1,977 kb, and the L50/N50 increased from 0.075 Mb in 6,213 scaffolds to 0.248 Mb in 1,887 scaffolds.

We further improved the Chicago-scaffolded assembly using 2 Hi-C chromosome conformation capture libraries from Dovetail (Lieberman-Aiden et al. 2009; Belton et al. 2012). Briefly, for each library, chromatin was fixed in place with formaldehyde in the nucleus and extracted. Fixed chromatin was digested with DpnII, 5′ overhangs were filled in with biotinylated nucleotides, and free blunt ends were ligated. After ligation, crosslinks were reversed and the DNA was purified from protein. Purified DNA was treated to remove biotin not internal to ligated fragments. The DNA was then sheared to approximately 350 bp mean fragment size, and sequencing libraries were generated using NEBNext Ultra enzymes and Illumina-compatible adapters. Streptavidin beads were used to isolate biotin-containing fragments before PCR enrichment of each library. Dovetail sequenced these libraries on an Illumina HiSeq X. The number and length of read pairs produced for each library were: 158 million, 2 × 150 bp for library 1; 194 million, 2 × 150 bp for library 2. Together, these Dovetail HiC library reads provided 87.47× physical coverage of the genome (10–10,000 kb). After assembly with HiRise, the N50/L50 of the assembly increased to 11.89 Mb in 25 scaffolds and a longest scaffold of 146,334 kb.

We obtained C-values for genome size estimation from Plant Cytometry Services of Didam, the Netherlands. We shipped fresh leaf tissue, and DNA content was estimated with flow cytometry relative to *Vinca minor* with both DAPI and PI staining.

### Linkage Mapping

We generated F_2_ linkage-mapping populations for both the XX/XY and XX/XY_1_Y_2_ cytotypes of 96 offspring each. The original parents were collected from Wesley Chapel, TX and Marion, SC, respectively (Pickup and Barrett 2013). For each cytotype, a single pair of wild individuals was crossed to generate F_1_ offspring, and a single pair of F_1_ offspring was crossed to generate F_2_ seeds. Although we did not karyotype the parents, recent work with the species suggests that the chromosome variation identified by Smith (1964) persists stably (Grabowska-Joachimiak et al. 2015). Seeds from F_1_ plants were sterilized in 5% (V/V) bleach for 1 min and then washed in running tap water and distilled water. Sterilized seeds were spread on wet filter paper in Petri dishes and incubated in the dark at 4 °C to germinate. After germination (usually within 2–3 weeks), we transplanted seedlings into 6-inch plastic pots filled with Promix soil and sand (3:1 ratio) with 300 ml of Nutricote (14:13:13, slow releasing fertilizer) for each 60 lbs of Promix soil. We grew seedlings in a glasshouse set for 22 °C daytime, 18 °C nighttime temperatures and 16-h day length at the University of Toronto, St. George Campus. We watered on alternate days and randomized pots twice weekly for uniform growing conditions and to avoid edge effects. We phenotyped plants for sex at onset of flowering. After plants were sexed, we collected approximately 30 mg of young and healthy leaf tissue from individual plants and flash froze it in liquid nitrogen for RNA extraction. Total RNA was extracted using Spectrum plant total RNA Kit (Sigma-Aldrich) according to manufacturer’s instructions. We sent RNA samples to Genome Quebec Innovation Centre (McGill University), Montréal, QC, Canada for library preparation and sequencing. Libraries were prepared using NEB mRNA stranded library preparation method and sequenced on two lanes of Illumina NovaSeq S2 PE100 (2 × 100) sequencing platform using 96 barcodes. A total of ∼6.25 billion reads (6,244,145,277) were generated, ranging from ∼13 to ∼104 million reads per sample with an average of ∼32.5 (32,521,589) and median of ∼28 million (28,041,358). Raw sequence has been deposited on the SRA under accession PRJNA638915 (embargoed until November 1, 2021 or publication).

We aligned samples to the *R. hastatulus* TX primary assembly using STAR 2-pass (STAR_2.5.3a [Dobin and Gingeras 2015]). We sorted alignments, assigned read groups, and marked PCR duplicates using PicardTools 2.18.21-SNAPSHOT (https://broadinstitute.github.io/picard/, last accessed November 3, 2020). We called variants with bcftools 1.9-67-g626e46b (Li et al. 2009; Danecek et al. 2014) mpileup and call, and filtered using bcftools view for minimum sample depth 10, minimum sample quality 10, minimum site quality 50, allele frequency between 0.1 and 0.9, and minimum 25 individuals called. For use in linkage mapping, we further filtered our variants using bcftools filter and vcftools (Danecek et al. 2011) to remove mislabeled individuals and sites with more than 5% missing data. For linkage mapping, we converted data from vcf to 012 format using vcftools 0.1.15 and transformed to csvr format using custom scripts. Scripts are available at https://github.com/joannarifkin/Rumex_genome/tree/master/Variant_calling (last accessed November 3, 2020). We generated a linkage map using the R package ASMAP 1.0-4 (Taylor and Butler 2017), which implements the minimum spanning tree algorithm in an R/qtl-compatible interface (Wu et al. 2008; Broman and Sen 2009). We removed two and three individuals from the XX/XY and XX/XY_1_Y_2_ mapping populations, respectively, because of apparent contamination. Individuals with high missing data and genetic clones were removed. We generated our XX/XY linkage map using only markers on the 100 biggest scaffolds of our draft assembly and 322 smaller scaffolds that we identified as sex-linked, which contain 1.2 Gb of sequence data. Our XX/XY_1_Y_2_ linkage map used only markers on the 100 biggest scaffolds of our draft assembly. We filtered our initial set of variants (25,010 XX/XY/25,544 XX/XY_1_Y_2_) to remove colocalized markers that had the same cM positions (leaving 2,255 XX/XY/2,103 XX/XY_1_Y_2_) and distorted markers (leaving 988 XX/XY and 877 XX/XY_1_Y_2_). We then constructed final maps from these markers for XX/XY and XX/XY_1_Y_2_, respectively (see supplementary table S1, Supplementary Material online, for progeny sample sizes). We used custom scripts to fuse colocalized markers into the map and to merge secondary linkage groups to maximize the number of scaffolds in the chromosome-scale assembly. Because we did not have sequence data from the parents or grandparents, we were unable to identify recombination events that occurred in the male or female parent and therefore could not estimate sex-specific recombination. We therefore present sex-averaged recombination rates. Scripts and supplementary methods are available at https://github.com/joannarifkin/Rumex_genome/tree/master/Map_construction (last accessed November 3, 2020).

For the XX/XY cytotype, we recovered five major linkage groups with over 100 markers each (108–276), as well as six minor fragmentary linkage groups (5–36 markers). The sizes of the major linkage groups ranged from 75.52 to 108.91 cM, with a total map length of 550.126. This is broadly consistent with expectations for a linkage map with five chromosomes. Recombination frequency (cM) roughly correlates with physical length (bp). We recovered four major linkage groups with over 100 markers each (103–258) for the XX/XY_1_Y_2_ cytotype, as well as seven minor fragmentary linkage groups (2–52 markers). The largest minor linkage group, LG11, was largely collinear with the sex chromosome (LG10), containing the same scaffolds and overlapping positions. The sizes of the major linkage groups ranged from 61.74 to 167.07 cM, with a total map length of 584.819 cM. We assessed recombination rates visually using Marey maps (Chakravarti 1991) relating physical position of markers along scaffolds to recombination position along chromosomes.

We used Chromonomer 1.09 (http://catchenlab.life.illinois.edu/chromonomer/, last accessed November 3, 2020) to relate our linkage map to our genome assembly. We conducted manual edits to our linkage map before using Chromonomer, to position scaffolds unique to minor linkage groups on major linkage groups based on physically nearby markers, and to remove minor linkage groups. We used a script from Nucleomics-VIB’s BioNano Tools (https://github.com/Nucleomics-VIB/bionano-tools, last accessed November 3, 2020) to convert Dovetail’s assembly table file into an .agp file. With Chromonomer, we were able to place 1.09 GB (65%) of the Dovetail assembly into five main pseudomolecules representing the major chromosomes. For downstream analyses, we created custom Python scripts using the .agp file output from Chromonomer to translate scaffold positions from our draft assembly to positions along the chromosomes. XX/XY_1_Y_2_-cytotype positions were converted to linkage group positions based on the XX/XY-cytotype map to facilitate ease of comparison between the two cytotypes (e.g., to identify inversions).

Linkage mapping in low recombination areas remains challenging. From both sequencing technologies (GBS and RNAseq), we found less genetic variation in low-recombination areas of the genome (supplementary fig. S4, Supplementary Material online), as seen previously in other systems (Nachman 2002). The diversity lowers faster in RNAseq than in the GBS data, likely because of the lower gene density (fig. 3). Our ability to infer genomic positions in the centric regions is thus limited by a shortage of both recombination events and variable markers in low-recombination areas, although the limited number of recombination events means that our marker density is still sufficiently high in these regions to have a robust average estimate. Because of the higher repeat content, even long-read sequence assembly remains challenging in low-recombination areas (Li et al. 2018). Although the reduced genetic variability in centric regions may lower our ability to estimate recombination rate from population data, as these estimates depend on both *N*_e_ and *r*, low genetic variability is unlikely to reduce our estimate of recombination from the cross. Without invoking double crossovers (which are not necessary to adequately account for the data elsewhere in the genome), missing markers could lead to a more gradual decline in recombination rate across a region but are unlikely to affect recombination rate estimates. Thus, our finding of large regions of low recombination across the *R. hastatulus* genome is likely to be robust to low marker density.

### Sex-Linked Variant Calling and Filtration

For RNA samples from Hough et al. (2014) and Beaudry et al. (2020), we aligned samples to the *R. hastatulus* XX/XY-cytotype primary assembly using STAR 2-pass 2.5.3a (Dobin and Gingeras 2015). We aligned reads for GBS samples from Beaudry et al. (2017) using NextGenMap 0.5.5 (Sedlazeck et al. 2013). For both alignments, we sorted the reads and assigned read groups using PicardTools 2.18.21-SNAPSHOT (https://broadinstitute.github.io/picard/, last accessed November 3, 2020). We marked PCR duplicates for RNAseq but not for GBS data. For both data sets, variants were called using bcftools 1.9-67-g626e46b mpileup as described above. We filtered the RNAseq data sets for minimum sample quality 20, minimum site quality 20, minor allele frequency >0.04, and no missing data. We filtered the GBS data set for minimum sample quality 20, minimum site quality 10, minimum mean depth of 6, minor allele frequency >0.05, and no more than 50% missing data. We converted data for windowed analyses from vcf to 012 format using vcftools. No individuals overlapped between the population RNAseq, linkage mapping RNAseq, and population GBS data. Scripts are available at https://github.com/joannarifkin/Rumex_genome/tree/master/Variant_calling (last accessed November 3, 2020).

We identified SNPs as showing X-linked, Y-linked, hemizygous, or autosomal segregation patterns, or male-only expression, in the cross data, the F_2_ data, and the population data using the 012 files described above and custom R scripts (available at https://github.com/joannarifkin/Rumex_genome/tree/master/Windowed_analyses, last accessed November 3, 2020) based on the segregation patterns described in Hough et al. (2014) in R version 3.6.3. We converted all sites to positions along chromosomes using custom scripts in Python 3.6.8 and 3.7 (available at https://github.com/joannarifkin/Rumex_genome/tree/master/Position_conversion, last accessed November 3, 2020) and the Chromonomer .agp file. We summed the different categories of sites across 500-kb windows using custom R scripts (available at https://github.com/joannarifkin/Rumex_genome/tree/master/Windowed_analyses, last accessed November 3, 2020).

### QTL Mapping and GWAS

We performed QTL mapping of sex as a binary phenotype using the scan1 function of R/qtl2 0.22 (Broman et al. 2019) using the F_2_ mapping population. We adjusted eta_max (the maximum value for the linear predictor in the model) downwards until the model was able to converge. We performed a permutation analysis to identify significance thresholds. We performed GWAS analysis in Gemma 0.98.1 (Zhou and Stephens 2012) using a likelihood ratio test for significance. Scripts are available at https://github.com/joannarifkin/Rumex_genome/tree/master/QTL_and_GWAS_mapping (last accessed November 3, 2020).

### Positioning of Previously Generated Divergence Data

To examine the divergence values from Hough et al. (2014) in the context of our new genome assembly, we used BLAST (Johnson et al. 2008) to align the previously generated transcriptome assembly to our new genome assembly. We retained BLAST hits that were ≥99% identical and converted positions using a custom Python and R scripts (scripts available at https://github.com/joannarifkin/Rumex_genome/tree/master/Position_conversion, last accessed November 3, 2020 and https://github.com/joannarifkin/Rumex_genome/tree/master/Windowed_analyses, last accessed November 3, 2020).

### Gene and Repeat Annotation

To annotate the genome, we used the gene modeling package BRAKER 2.1.2 (Hoff et al. 2019) to predict gene positions. We first created a repeat database with repeatModeler 1.0.11 (Smit et al. 2013) using NCBI’s search engine and parameter -pa set to 3. The genome was then masked for repetitive elements using RepeatMasker 4.0.7 (Smit et al. 2013). Next, BRAKER called AUGUSTUS 3.3.3 (Stanke and Morgenstern 2005) to refine gene prediction based on annotations in other organisms. For AUGUSTUS, we used the *Arabidopsis thaliana* gene models (Stanke and Morgenstern 2005). We further refined AUGUSTUS’s models using four XX/XY-cytotype RNAseq data sets: two sequenced from pollen and one each from male and female flower bud tissue (Sandler et al. 2018). Alignment of the RNAseq data to the genome assembly was performed as above (see Sex-Linked Variant Calling and Filtration). BRAKER iteratively improved these gene models over four rounds of AUGUSTUS, which reduced the number of false positives due to repetitive elements. Alignment of the RNAseq data was performed as above (see Sex-Linked Variant Calling and Filtration). BRAKER returned a set of 84,408 predicted genes.

To further remove repetitive elements, we aligned the RNAseq data sets and the whole-genome sequencing data from Beaudry et al. (2017) to the predicted transcriptome. For RNAseq, we used STAR (as above), whereas for the alignment of genomic reads we used NextGenMap (Sedlazeck et al. 2013) with default parameter settings. All reads were sorted using Picard’s SortSam and then reads were indexed and counted using Samtools’s index and idxstats, respectively. Loci with >log(0.05) coverage and <0.03 reads per kilobase of transcript per million mapped reads (RPKM) were discarded from the annotation. After filtering, we retained 42,994 annotated genes, 33,309 of which were placed on the five major linkage groups. We verified the gene annotation against the BUSCO v4.0.5 (Seppey et al. 2019) set of conserved eukaryote genes, yielding 33% complete and 19% fragmented, and land plant (Viridiplantae) genes, yielding 30% complete and 22% fragmented.

We identified repeats using RepeatMasker (Smit et al. 2013). For windowed analysis, we determined both the number of start sites and the proportion of sequence contained within either repetitive elements or coding sequences in windows across the genome. Gene and repeat positions were converted from scaffold positions to linkage group positions using custom Python scripts and windowed using custom R scripts.

## Supplementary Material

Supplementary data are available at *Molecular Biology and Evolution* online.

## Supplementary Material

msaa271_Supplementary_Data
