# Supplementary material for: Widespread Recombination Suppression Facilitates Plant Sex Chromosome Evolution
Source: Mol Biol Evol. 2020 Oct 23;38(3):1018–30. doi: 10.1093/molbev/msaa271 (PMC7947811; doi:10.1093/molbev/msaa271)
Supplement: msaa271_Supplementary_Data [file msaa271_supplementary_data.pdf]

Supplementary Materials

Supplementary Tables

Table S1. C-values calculated from two XX/XY-cytotype individuals [an F_3 Male (ID: TxTx P5 MG) and F_3 Female (ID: TxTx P5 MG)] of *Rumex hastatulus*

	XX/XY
Female	1.8
Male	1.99

Table S2. Counts of *Rumex hastatulus* individuals of each sex and cytotype sequenced for the linkage map.

	TX (XX/XY)	NC (XX/XY_1Y_2)
Male	53	40
Female	43	56

Table S3. Major linkage groups in both maps of *Rumex hastatulus*

LG ID numbers after Smith 1964

TX (XX/XY)

Description	LG ID	Number of independent markers (colocalized markers)	Length (cM)	Length (bp)	Length (μm, Grabowska-Joachimiak 2015)
Metacentric autosome	A1	192 (1570)	101.022	344,497,533	3.8
Metacentric autosome	A2	276 (3241)	108.910	260,405,700	5.02
Neo-sex chromosome	A3	108 (338)	95.789	175,013,671	2.35
Sub-metacentric autosome	A4	119 (510)	48.148	158,234,227	2.81

Sex chromosome	XY	187 (1186)	75.521	150,617,407	3.4 (X)<br>5.89 (Y)
		882 (6845)	429.39	1088768538	
NC (XX/XY_1Y_2)					
Description	LG ID	Number of independent markers (colocalized markers)	Length (cM)	Length (bp)	Length (physical, as percentage of karyotype, Smith 1964)
Metacentric autosome B	A1	184 (645)	162.541		3.67
Metacentric autosome A	A2	258 (2576)	167.069		4.98
Sub-metacentric autosome	A4	103 (552)	60.524		2.2

Sex chromosome	XYY	250 (1270)	137.695		5.36 (X)<br>4.22 (Y1)<br>3.77 (Y2)
		537 (5043)	527.829		

7

Table S4. Sex-linked SNPs of *Rumex hastatulus* based on Hough et al. (2014) pedigree data. TX = XX/XY, NC = XX/XY_1Y_2 .

	Y-segregating (TX)	Y-segregating (NC)	X-segregating (TX)	X-segregating (NC)	Hemizygous (TX)	Hemizygous (NC)	Autosomal (TX)	Autosomal (NC)
A1	14	57	5	7	1	1	3894	1692
A2	3	137	1	18	13	5	1700	3950
A3	12	1172	11	763	6	7	1614	410
A4	4	59	0	33	2	0	1173	1691
X	1208	980	439	456	50	79	420	348
Not placed	1048	1110	143	173	317	345	1681	1714

Table S5. Sex-linked SNPs of *Rumex hastatulus* based on Hough et al. (2014) and Beaudry et al. (2019) population data. TX = XX/XY, NC = XX/XY_1Y_2.

	XY-fixed (TX)	XY-fixed (NC)
A1	17	16
A2	4	76
A3	7	390
A4	5	3
X	1048	583
Not placed	1403	1254

Table S6. Coefficients of linear models relating base pairs to centimorgans in windows on sections of the sex chromosome and A3 in *Rumex hastatulus*.					
Cytotype	Chromosome	Block	Window size (bp)	Mean SNPs per window	Mean coefficient of by-window linear models (cM/bp)
	Sex chromosome	Pseudoautosomal and sex-linked recombining region (first 50Mb)	50000	4.415	4.02×10^{-7}
			100000	5.5	1.13×10^{-6}
			500000	11.386	1.43×10^{-6}
			1000000	19.088	1.29×10^{-6}
			5000000	72.111	1.24×10^{-6}
		Sex-linked region (second 50Mb)	50000	2.563	-1.23×10^{-17}
			100000	2.716	-1.28×10^{-17}
			500000	4.233	-1.16×10^{-17}
			1000000	5.871	-4.24×10^{-18}
			5000000	26	-8.25×10^{-8}

XX/XY	A3 (neo-sex chromosome)	XX/XY-specific low-recombination region (final 50Mb)	50000	1.556	-6.57×10^{-18}
			100000	1.68	-5.40×10^{-18}
			500000	2.1	3.51×10^{-7}
			1000000	2.8	5.71×10^{-7}
			5000000	8.4	4.03×10^{-7}
		High-recombination region / putative XX/XY_1Y_2 pseudoautosomal region (penultimate 50Mb)	50000	2.778	2.72×10^{-17}
			100000	3.175	1.25×10^{-6}
			500000	4.878	1.83×10^{-6}
			1000000	6.897	1.20×10^{-6}
			5000000	22.222	8.09×10^{-7}
		Shared low-recombination region / putative XX/XY_1Y_2 sex-linked region (second 50Mb)	50000	1.5	1.44×10^{-16}
			100000	1.5	1.44×10^{-16}
			500000	1.5	1.44×10^{-16}
			1000000	1.5	1.44×10^{-16}
			5000000	1.8	2.84×10^{-7}
			50000	3.243	-0.001

XX/XY_1Y_2	Sex chromosome	Pseudoautosomal and sex-linked recombining region (first 50Mb)	100000	3.948	-0.001
			500000	7.567	-2.22 x 10^{-5}
			1000000	11.641	-3.08 x 10^{-5}
			5000000	45.4	2.27 x 10^{-7}
		Sex-linked region (second 50Mb)	50000	1.827	-3.01 x 10^{-17}
			100000	2	-2.26 x 10^{-17}
			500000	2.8	-1.61 x 10^{-17}
			1000000	2.8	-1.61 x 10^{-17}
			5000000	6	-1.15 x 10^{-17}
	A3 (neo-sex chromosome)	High-recombination region / putative XX/XY_1Y_2 pseudoautosomal region (final 50Mb)	50000	3.144	5.34 x 10^{-5}
			100000	4	5.75 x 10^{-5}
			500000	9.576	2.39 x 10^{-6}
			1000000	16.205	1.92 x 10^{-6}
			5000000	63.2	-1.22 x 10^{-7}
		Shared low-recombination region / putative	50000	2.355	1.39 x 10^{-17}
			100000	2.517	1.27 x 10^{-17}
			500000	3.65	3.16 x 10^{-18}

		XX/XY_1Y_2 sex-linked region (penultimate 50Mb)	1000000	4.563	3.62×10^{-18}
			5000000	14.6	1.28×10^{-21}

Supplementary Figures

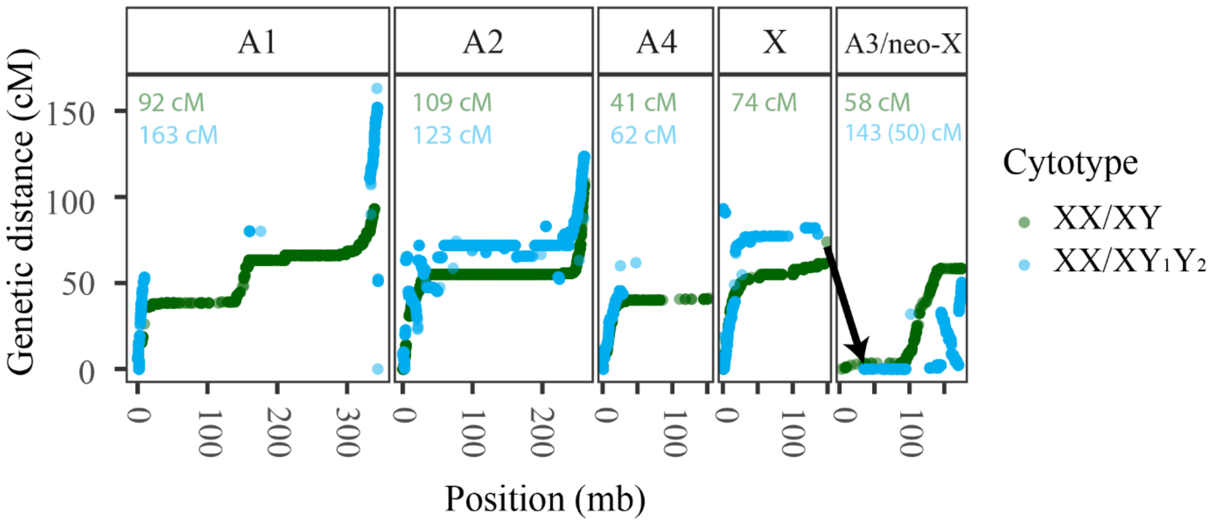

Figure S1. Marey map relating linkage map sex-averaged recombination position (cM) to physical genetic position in the XX/XY-cytotype genome assembly (Mb; panels are XX/XY-cytotype chromosomes) for the XX/XY cytotype (green) and XX/XY_1Y_2 cytotype (blue) of *Rumex hastatulus*. XX/ XY_1Y_2cytotype map on the neo-X has been artificially reset to zero, to ease comparison with map distances along A3 in XX/XY cytotype (as shown by the black arrow). Total map length for each chromosome in top right corner of each panel, XX/XY cytotype (green) and XX/XY_1Y_2 cytotype (blue), with the map length specific to the neo-X in brackets in the A3/neo-X panel.

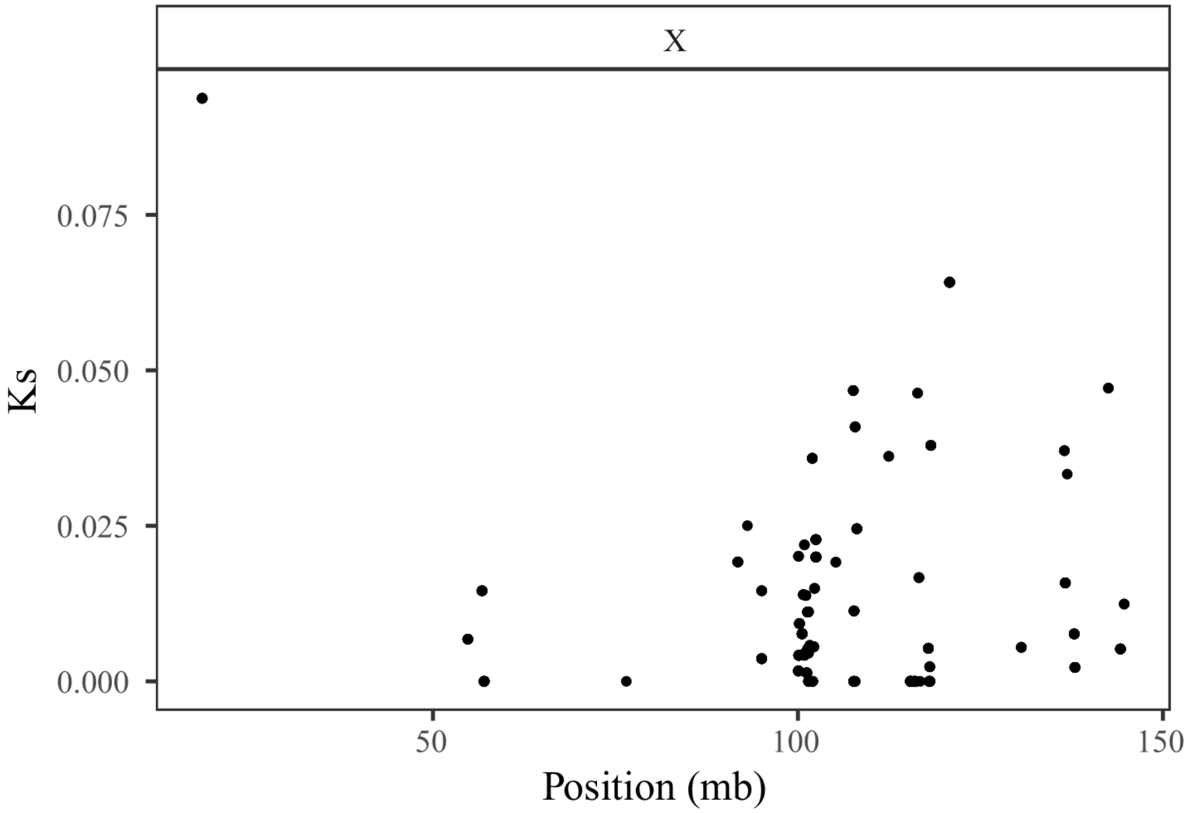

Figure S2. Synonymous site divergence (Ks) between the X and Y chromosome along the shared X chromosome of *Rumex hastatulus* from (Hough et al. 2014).

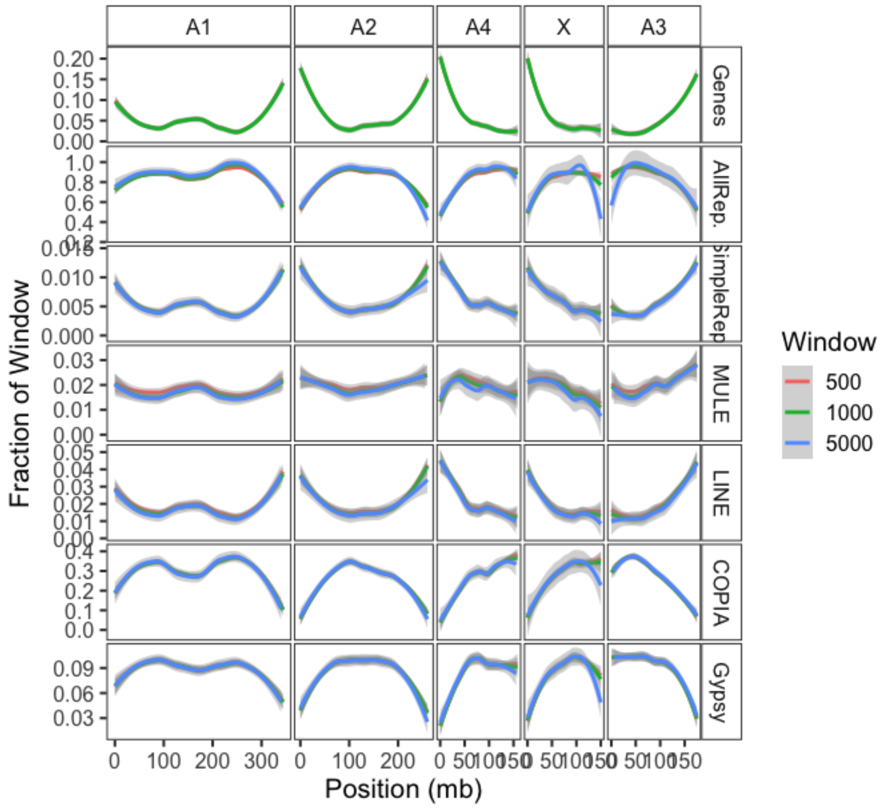

Figure S3. Genome content across the chromosomes of *Rumex hastatulus*, measured as proportion of window across varying window sizes (red: 500 kilobases; green: 1000 kilobases; blue: 5000 kilobases). Panels show repeat-filtered gene density, density of all repetitive elements (“AllRep.”), simple repeat density (“simpRep”), MULE element density, LINE element density, COPIA element density, and Gypsy element density.

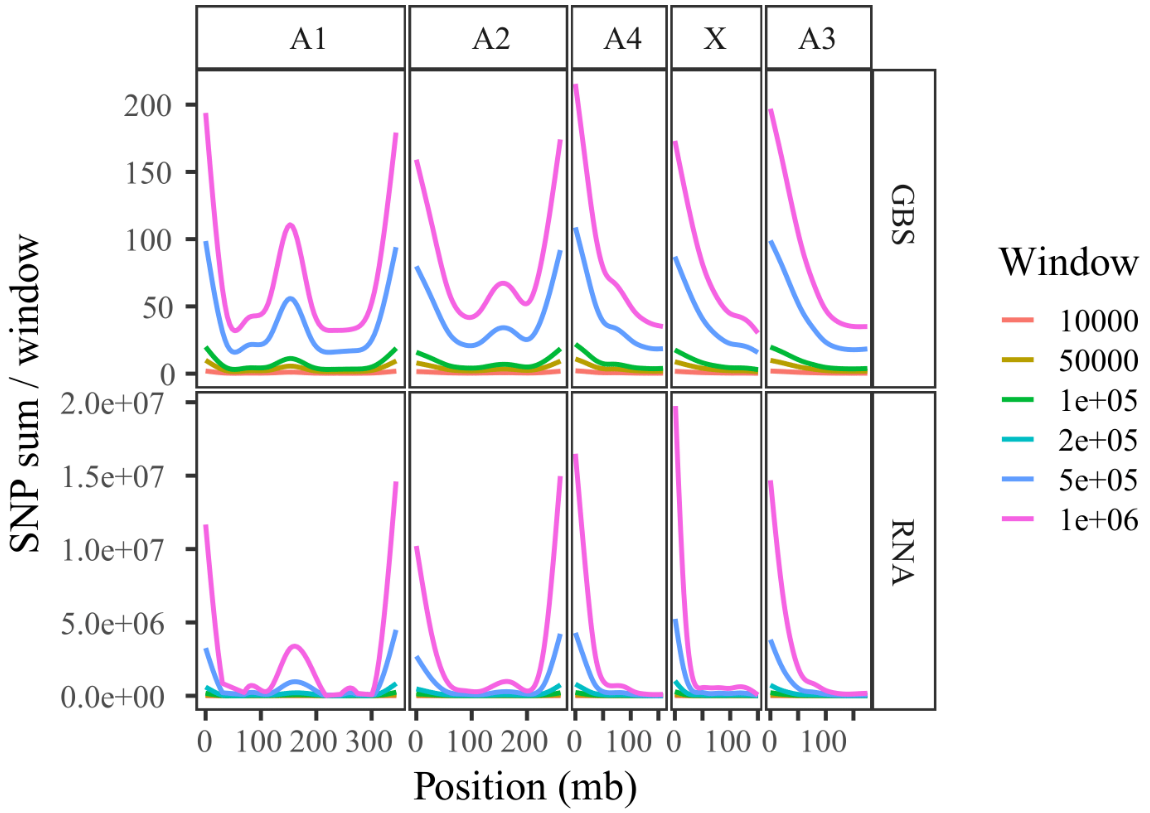

Figure S4. SNP density for GBS and RNA data sets summed across the five chromosomes of *Rumex hastatulus* in windows of varying sizes (colour). Window sizes are in base pairs.
